# Supplementary material for: SARS-CoV-2 neutralizing human recombinant antibodies selected from pre-pandemic healthy donors binding at RBD-ACE2 interface
Source: Nat Commun. 2021 Mar 11;12:1577. doi: 10.1038/s41467-021-21609-2 (PMC7952403; doi:10.1038/s41467-021-21609-2)
Supplement: Supplementary file 1 — Supplementary Information [file 41467_2021_21609_MOESM1_ESM.pdf]

Supplementary Information

SARS-CoV-2 neutralizing human recombinant antibodies selected from pre-pandemic healthy donors binding at RBD-ACE2 interface

Bertoglio *et al.*

Supplementary Figure 1 Schematic overview of the expressed variants of the Spike SARS-CoV-2 protein.

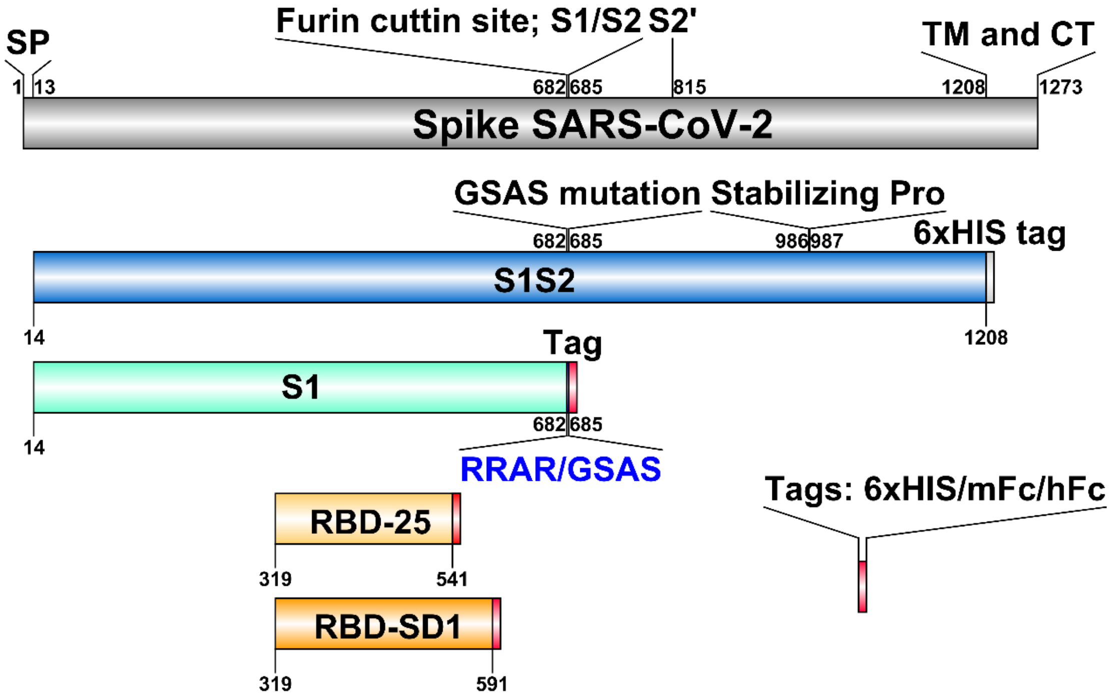

Supplementary Figure 2 SEC data of antigen produced in insect High Five cells (A-C) and mammalian Expi293F cells (D-E). The table (F) indicates the most likely conformation of the protein due to the retention volume of the peaks with the corresponding area in percentage. (*Furin site is present). Data shows a representative of two measurements.

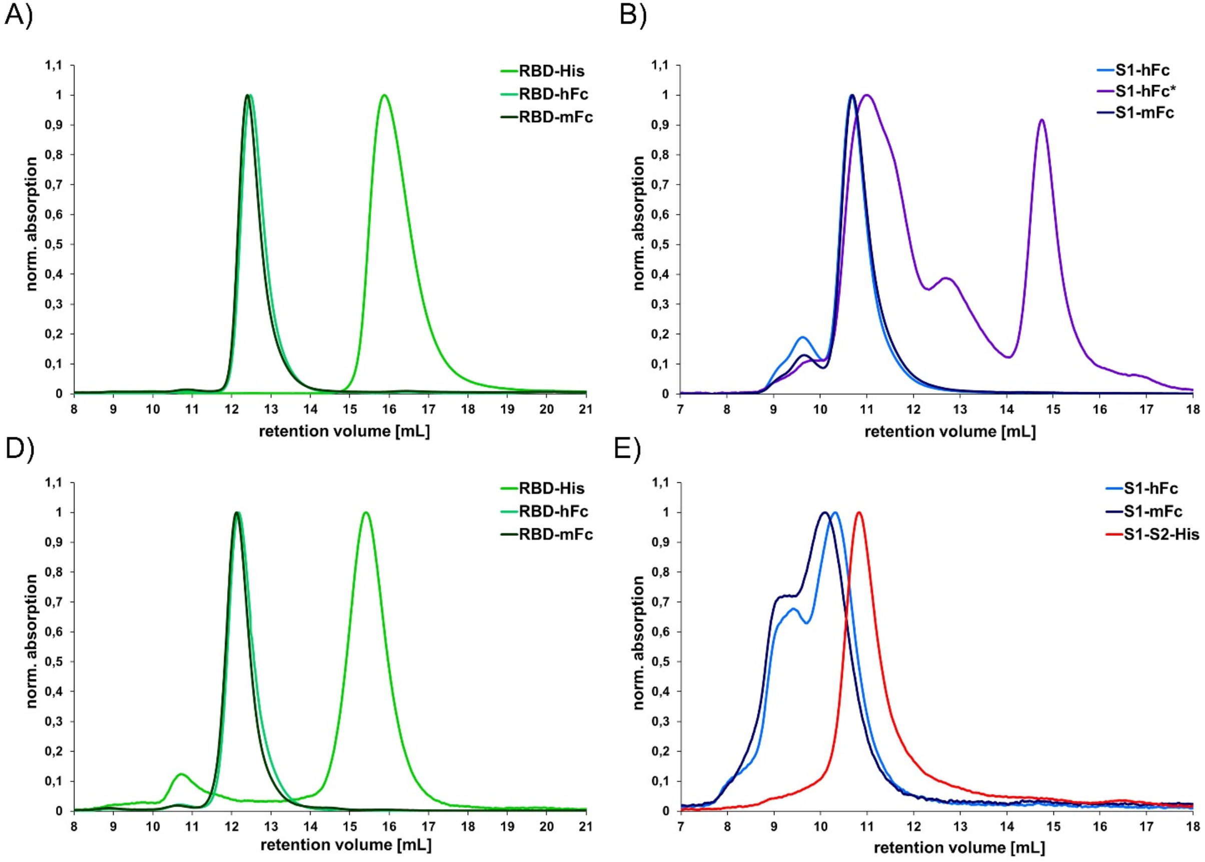

F)

Antigen	Main Peak	High Five cells Vol [mL]	High Five cells Area [%]	Expi293F cells Vol [mL]	Expi293F cells Area [%]
RBD-His	Monomer	15.9	99	15.4	93
RBD-hFc	Dimer	12.5	100	12.2	98
RBD-mFc	Dimer	12.4	99	12.1	99
S1-His	Monomer	12.7	100	nd	nd
S1-hFc	Aggregate?	9.6	16	9.3	31
	Dimer	10.7	84	10.3	68
S1-hFc*	Dimer	10.9	58	nd	nd
	S1-hFc-hFc	12.8	9	nd	nd
	hFc Dimer	14.8	33	nd	nd
S1-mFc	Aggregate?	9.7	11	9.1	27
	Dimer	10.7	89	10.1	73
S1-S2-His	Aggregate?	9.2	23	-	-
	Trimer	9.8	50	-	-
	Monomer	11.2	27	10.8	100

Supplementary Figure 3 Flow cytometry inhibition analysis A) Gating strategy B) Analysis of spike S1-S2 trimer (50 nM in relation to monomer) binding to living cells expressing ACE2 blocked by 1500 nM antibodies and C) of RBD-mFc (10 nM in relation to monomer) binding to living cells expressing ACE2 blocked by 100 nM antibody. The antibodies STE72-1G5, STE72-4C10, STE72-8E1 and STE73-6C8 were used as example. The background control are transfected ACE2 cells and the reference are ACE2 positive cells incubated with labeled spike protein. Data shows a representative of the flow cytometry inhibition experiments.

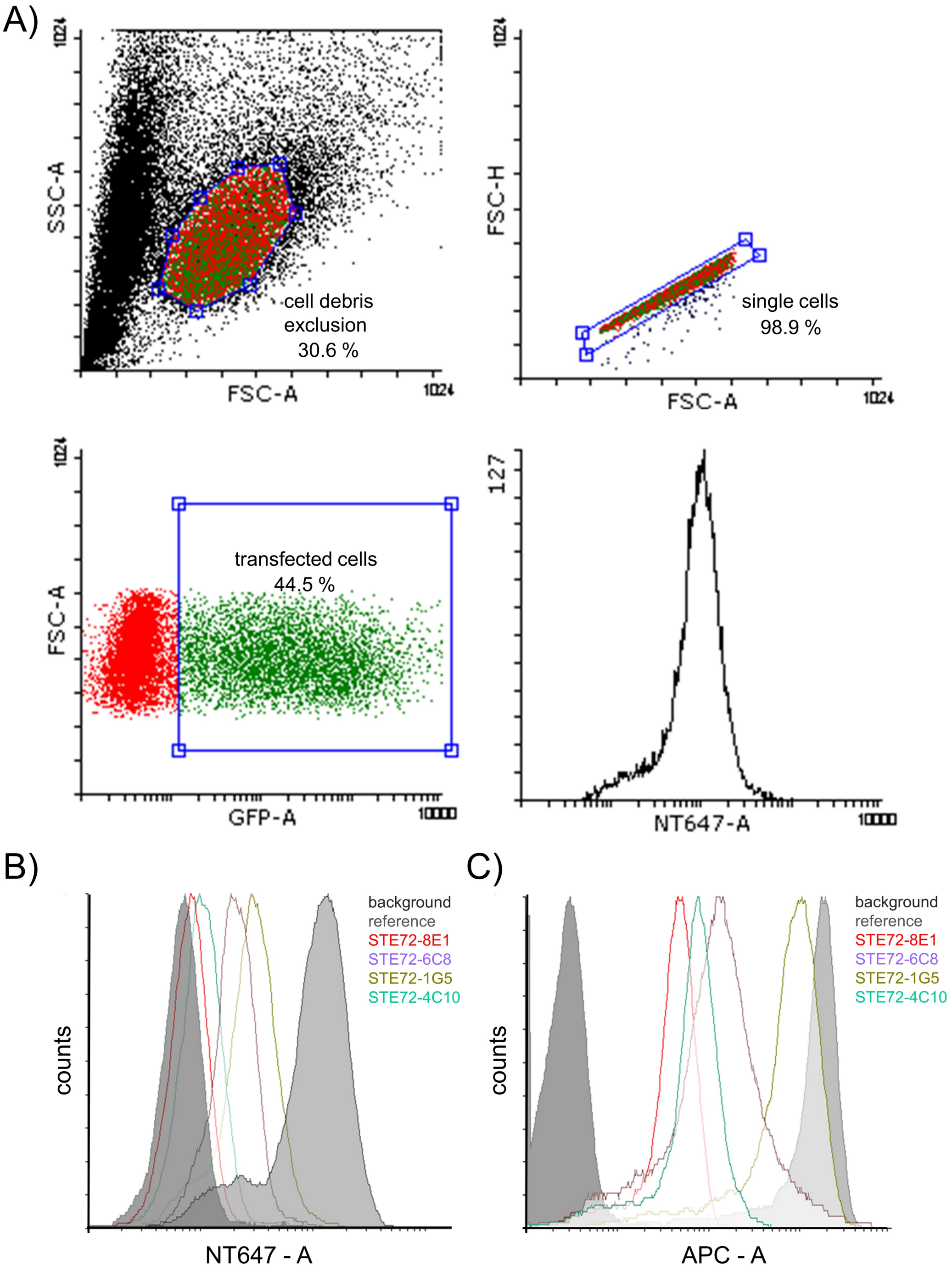

Supplementary Figure 4 VH and VL V-genes of 17 inbiting and neutralizing scFv-Fc. The CDRs are given according to the IMGT nomenclature (http://www.imgt.org/IMGTScientificChart/Numbering/IMGTIGVLsuperfamily.html) using VBASE2 (www.vbase2.org).

Antibody name	VH amino acid sequence<br>**CDR1**, ***CDR2***, **CDR3**	VL amino acid sequence<br>**CDR1**, ***CDR2***, **CDR3**
**STE70-1E12**	QMQLVQSGAEVKKPGASVKVSCKAS**GYTFTGYY**MHWVRQAP<br>GQGLEWMGR***INPNSGGT***NYAQKFQGRVTMTRDTSTSTAYME<br>LSRLRSDDTAVYYC**VRDGYNFNNWFDP**WGQGTLVTVSS	NFMLTQPHSVSGSPGETITISCTGS**GGSIASNY**VQWYRQRP<br>GSAPTIVIY***EDT***QRPSGVPDRFSGSIDSSSNSASLTISGLK<br>TEDEADYYC**QSSESTNHVM**FGGGSKLTVL
**STE72-1B6**	QVKLVQSGGGLVQPGGSLRLSCAAS**GFTFSSYA**MSWVRQAP<br>GKGLEWVSG***ISGSGGST***YYADSVKGRFTISRDNSKNTLYLQ<br>VNSLRAEDTAVYYC**AKAPYGDFRGLWYFDY**WGQGTLVTVSS	DIVMTQSPFSVSASVGDRVTISCRAS**QDIRSW**LAWYQQKPG<br>KAPQLLIY***DAS***SLQSGVPSRFSGSGSGTDFTLTISSLQPED<br>FATYYC**QQADSVPLT**FGGGTKVEIQ
**STE72-1G5**	QVQLVQSGAEVKKPGASVKVSCKAS**GGTFSSYA**ISWVRQAP<br>GQGLEWMGG***IIPIFGTA***NYAQKFQGRVTITADESTSTAYME<br>LSSLRSEDTAVYYC**AVGGVQLWLTY**WGQGTLVTVSS	ETTLTQSPGALSLSPGERATLSCRVS**QSLSSNS**LAWYQQKP<br>GQAPRLLIY***DTS***IRATGIPDRFSGSGSGTDFTLTISRLEPE<br>DFAVYYC**HKYGSWPRT**FGQGTKVEV
**STE72-4C10**	EVQLQESGGGVVQPGRSLRLSCAAS**GFTFSSYA**MHWVRQAP<br>GKGLEWVAV***ISYDGSNK***YYADSVKGRFTISRDNSKNTLYLQ<br>MNSLRAEDTAVYYC**AREYSSSWYGLGAFDI**WGQGTMVTVSS	DIRLTQSPSSLSASVGDRVTITCRAS**QNIHYY**LNWYQQKPG<br>KAPSLLVY***GAS***SLQSGVPSRFSGSGSGTEYTLTISSLQPED<br>FATYFC**QQSFKSPAT**FGQGTKVDIK
**STE72-4E12**	QVQLVQSGAEVKKPGASVKVSCKAS**GYTFTSYY**MHWVRQAP<br>GQGLEWMGI***INPSGGST***SYAQKFQGRVTMTRDTSTSTVYME<br>LSSLRSEDTAVYYC**ARDLSGGLDY**WGQGTLVTVSS	ETTLTQSPATLSVSPGERVTLACRAG**QTISSK**LAWYRQKPG<br>QAPSLLIY***DAS***TRATGVPARFSGSGSGTEFTLTISSLQSED<br>FAVYYC**QQYSDWPPT**FGQGTKVEIK
**STE72-8A2**	QVQLVQSGAEVKKPGASVKVSCKAS**GYTFTSYG**ISWVRQAP<br>GQGLEWMGW***ISAYNGNT***NYAQKLQGRVTMTTDTSTSTAYME<br>LRSLRSDDTAVYYC**ARFFYDSSGYSTDY**WGQGTLVTVSS	DIRLTQSPSSLSASVGDRVTITCQAS**QDISNY**LNWYQQKPG<br>KAPKLLIY***DAS***NLETGVPSRFSGSGSGTDFTFTISSLQPED<br>IATYYC**QQYDNLPRT**FGGGTKVEIK
**STE72-8A6**	QVQLVQSGAEVKKPGASVKVSCKAS**GYTFTSYG**ISWVRQAP<br>GQGLEWMGW***ISAYNGNT***NYAQKLQGRVTMTTDTSTSTAYME<br>LRSLRSDDTAVYYC**ARFFYDSSGYSTDY**WGQGTLVTVSS	DIVMTQSPSTLSASVGDRVTISCRAS**QNIGTW**LAWYQQKPG<br>KAPKLLIS***KAS***SLESGVPSRFSGSGSGTDFTLTISSLQPDD<br>FATYYC**QQYDSYLFT**FGQGTKLEIR
**STE72-8E1**	QVQLQEWGPGLVQPSETLSLTCTVS**GGSISSGGYY**WSWIRQ<br>HPGKGLEWIGR***IYPSGST***NYNPSLKSRVTMSVDTSENQFSL<br>KLSSVTAADTAVYYC**ARGGPKRSGSPFDV**WGQGTMVTVSS	EVVMTQSPSTLSASVGDRVTITCRAS**QTFGPW**LAWYQQKPG<br>QAPKLLIY***MAS***ELESGVPSRFSGSGSGTEFTLSISSLQPDD<br>FATYYC**QQYNTFSPWT**FGQGTKVDIK
**STE72-2G4**	QVQLVQSGAEVKKPGASVKVSCKAS**GYTFTSYG**ISWVRQAP<br>GQGLEWMGW***ISAYNGNT***NYAQKLQGRVTMTTDTSTSTAYME<br>LRSLRSDDTAVYYC**ARFFYDSSGYSTDY**WGQGTLVTVSS	ETTLTQSPAFMSVTPGDRAILSCRAS**QSVDSH**LAWYHQKPG<br>QPPRLLIY***GAS***TRATGIPARFSGSRSGTEFTLTISGLQSED<br>LGVYYC**QQYASWPPGYN**FGQGTKLEIK

**STE73-2B2**	QVQLVQSGAEVKKPGASVKVSCKAS**GYTFTGYY**MHWVRQAP<br>GQGLEWMGW***INPNSGGT***NYAQKFQGRVTITRDTSASTAYME<br>LSSLRSEDTAVYYC**ARVSGWYFGAFDI**WGQGTMVTVSS	LPVLTQPHSLSESPGKTVNISCTGS**SGSIASNY**VQWYQQRP<br>GSAPTTVIY***EDS***HRPSGVPDRFSGSIDSSSNSASLSISGLK<br>TEDEADYYC**QSYDSSNNQWV**FGGGTKLTVL
**STE73-2C2**	QVQLVESGGGLVQPGGSLRLSCAAS**GITVSSNY**MSWVRQAP<br>GKGLEWVSV***IYSGGST***FYADSVKGRFTISRDNSKNTLYLQM<br>NSLRAEDTGVYYC**ARGHDNLDY**WGQGTLVTVSS	LPVLTQPHSVSESPGKTVIISCTGS**SDSIASNY**VQWYQQRP<br>GSAPTTVIY***EDN***KRPSGVPDRFSGSTDSSSNSASLTISGLK<br>TEDEAEYYC**QSYDSNNHWV**FGGGTKLTVL
**STE73-2E9**	QVQLVQSGAEVKKPGASVKVSCKAS**GYTFTSYG**ISWVRQAP<br>GQGLEWMGW***ISAYNGNT***NYAQKLQGRVTMTTDTSTSTAYME<br>LRSLRSDDTAVYYC**ARGKFDY**WGQGTLVTVSS	QSVLTQPPSVSEAPSQRVTISCSGS**SSNIGNNA**VNWYQQLP<br>GKAPKLLIY***YDD***VLPSGVSDRFSGSKSGTSASLAISGLQSG<br>DEADYYC**AAWDDSLSGVV**FGGGTKLTVL
**STE73-2G8**	QVQLVQSGGGLVKPGGSLRLSCAAS**GITVSSNY**MSWVRQAP<br>GKGLEWVSI***LYSGGTT***FYTDSVKGRFTISRDNSKNTLHLQM<br>NSLRAEDTAVYYC**ARWSGTYYDY**WGQGTLVTVSS	SSELTQDPAVSVALGQTVRITCQGD**SLRSYY**ASWYQQKPGQ<br>APVLVIY***GKN***NRPSGIPDRFSGSSSGNTASLTITGAQAEDE<br>ADYYC**NSRDSTNDPVL**FGAGTKLTVL
**STE73-6B10**	QVQLVQSGAEVKKPGASVKVSCKAS**GYTFTSYG**ISWVRQAP<br>GQGLEWMGW***INPNSGGT***NYAQKFQGRVTMTRDTSISTAYME<br>LSRLRSDDTAVYYC**ARDRLRYGDSGSYYYYGMDV**WGQGTTV<br>TVSS	QSALTQPRSVSGSPGQSVTISCTGT**SSDVGGYNY**VSWYQQH<br>PGKAPKLLIS***EVT***RRPSGVPDRFSGSKSGNTASLTVSGLQT<br>EDEADYYC**GSYAGSNTWV**FGGGTKLTVL
**STE73-6C1**	QVQLVQSGGGVVQPGRSLRLSCAAS**GFTFSSYA**MHWVRQAP<br>GKGLEWVAV***ISYDGSNK***YYADSVKGRFTISRDNSKNTLYLQ<br>MNSLRAEDTAVYYC**ARSYVGGMDV**WGQGTTVTVSS	QLVLTQSPSVSGAPGQSVTISCTGT**DSNIGAGYN**VHWYQQL<br>PGAAPKLIIS***YTD***NRPSGVPDRFSGSKSGTSASLAITGLQA<br>EDEADYYC**QSSDRTLVGSL**FGTGTKVTVL
**STE73-6C8**	QVQLQQSGAEVKKPGSSVKVSCKAS**GGTFSSYA**ISWVRQAP<br>GQGLEWMGR***IIPILGIA***NYAQKFQGRVTITADKSTSTAYME<br>LSSLRSEDTAVYYC**ARSIAALNWFDP**WGQGTLVTVSS	NFMLTQPHSVSESPGKTVTIPCTGS**RGSIANNY**VQWYRQRP<br>GRAPTTVIF***EDN***QRPSGVPDRFSGSIDRSSNSASLTISGLE<br>TEDEADYYC**QSYDGSNVI**FGGGTKLTVL
**STE73-9G3**	QVQLQESGGGLVQPGGSLRLSCAAS**GFTFSSYA**MSWVRQAP<br>GKGLEWVSA***ISGSGGST***YYADSVKGRFTISRDNSKNTLYLQ<br>MNSLRAEDTAVYYC**ARDLVLGSGSSND**WGRGTLVTVSS	QPVLTQPPSVSGAPGQSVTISCTGT**DSNIGAGYN**VHWYQQL<br>PGAAPKLIIS***YTD***NRPSGVPDRFSGSKSGTSASLAITGLQA<br>EDEADYYC**QSSDRTLVGSL**FGTGTKVTVL

Supplementary Figure 5 Flow cytometry inhibition analysis A) Comparison of the inhibition of spike protein and RBD by flow cytometry on ACE2 positive Expi293F cells using 1000 nM scFv-Fc and 50 nM spike protein, respectively RBD (20:1 ratio). B) Inhibition of RBD binding in comparison of Expi293F transiently expressing ACE2 and Calu-3 cells. C) Inhibition of spike S1-S2 binding in comparison of Expi293F transiently expressing ACE2 and Calu-3. As negative control, the antibody SH1351-C1 was used. Data shows single measurements.

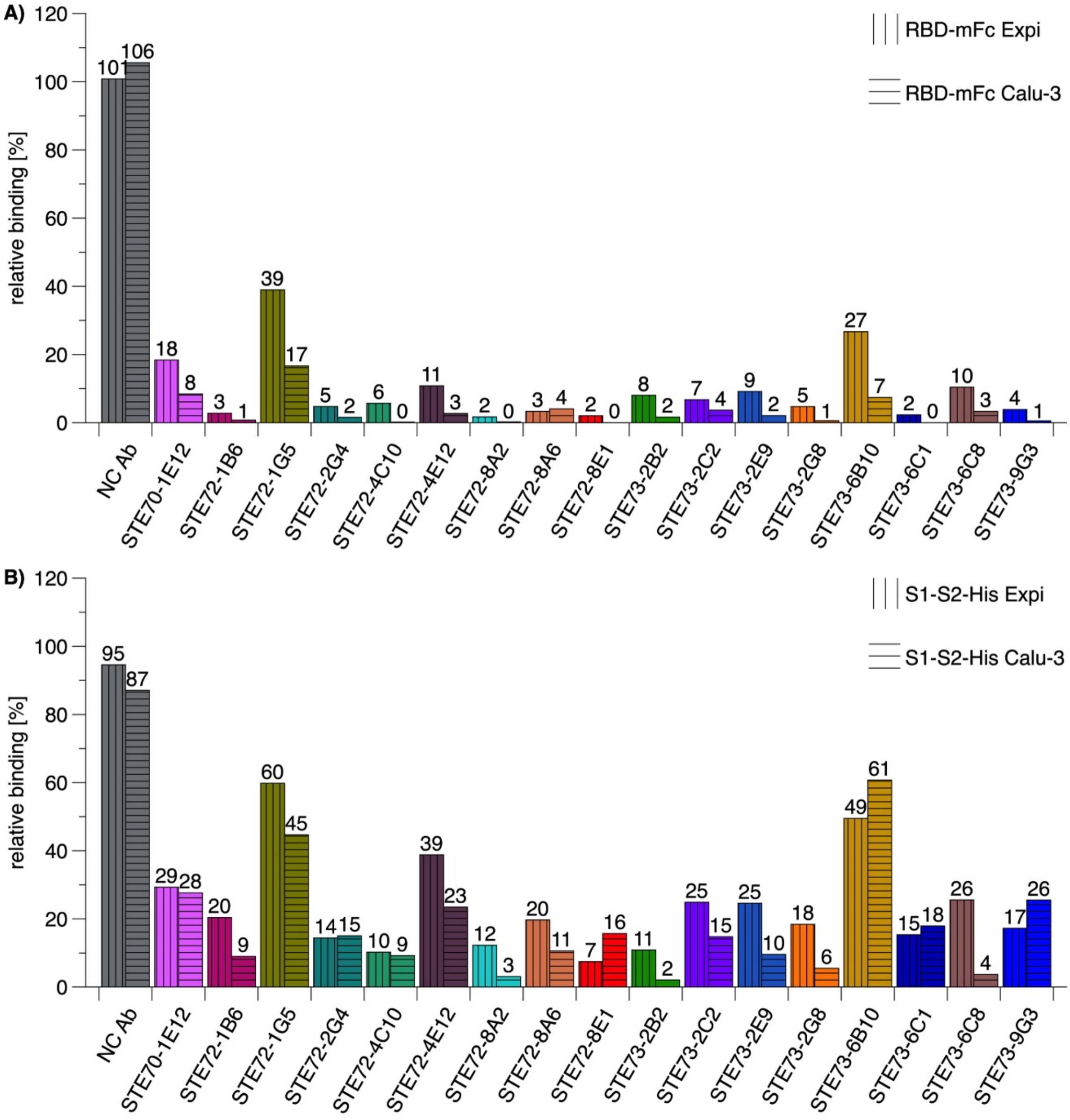

Supplementary Figure 6 Inhibition of SARS-CoV-2 spike protein binding to cells by antibody combinations. Flow cytometry analysis to determine the inhibition efficacy of antibody combinations on ACE2 positive cells and 50 nM S1-S2 and 1500 nM as a single antibody, respectively 750 nM of each antibody in a combination. Data shows single measurements.

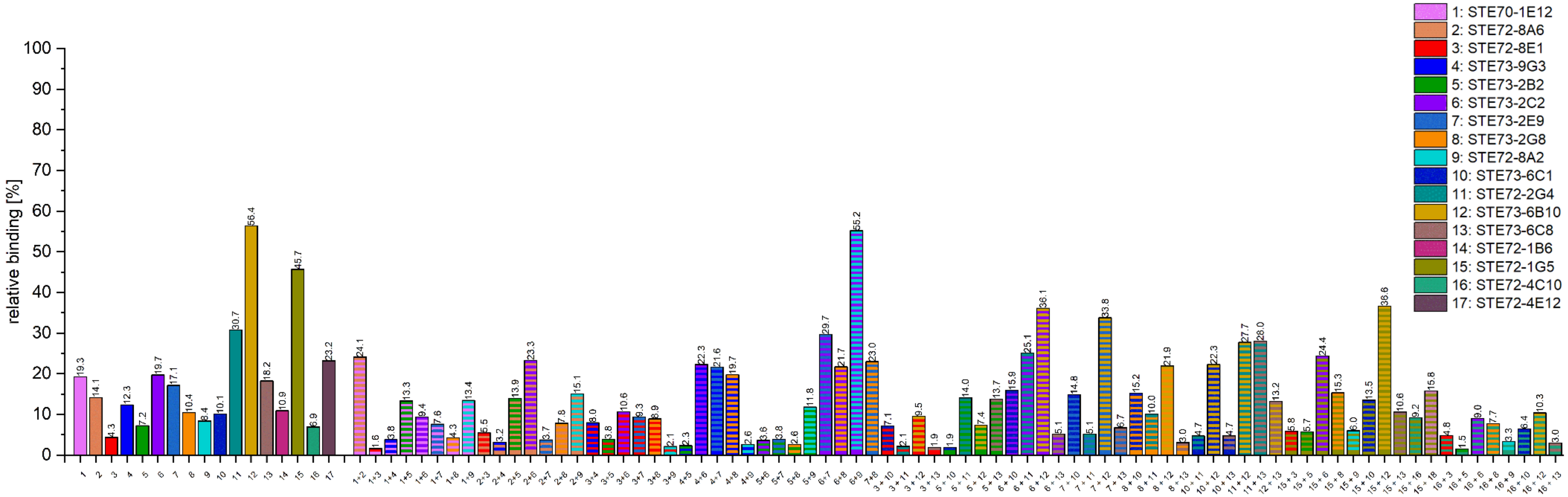

Supplementary Figure 7 Binding of the IgGs to RBD, S1 and S1-S2 in ELISA. Experiments were performed in duplicate and mean values are given.

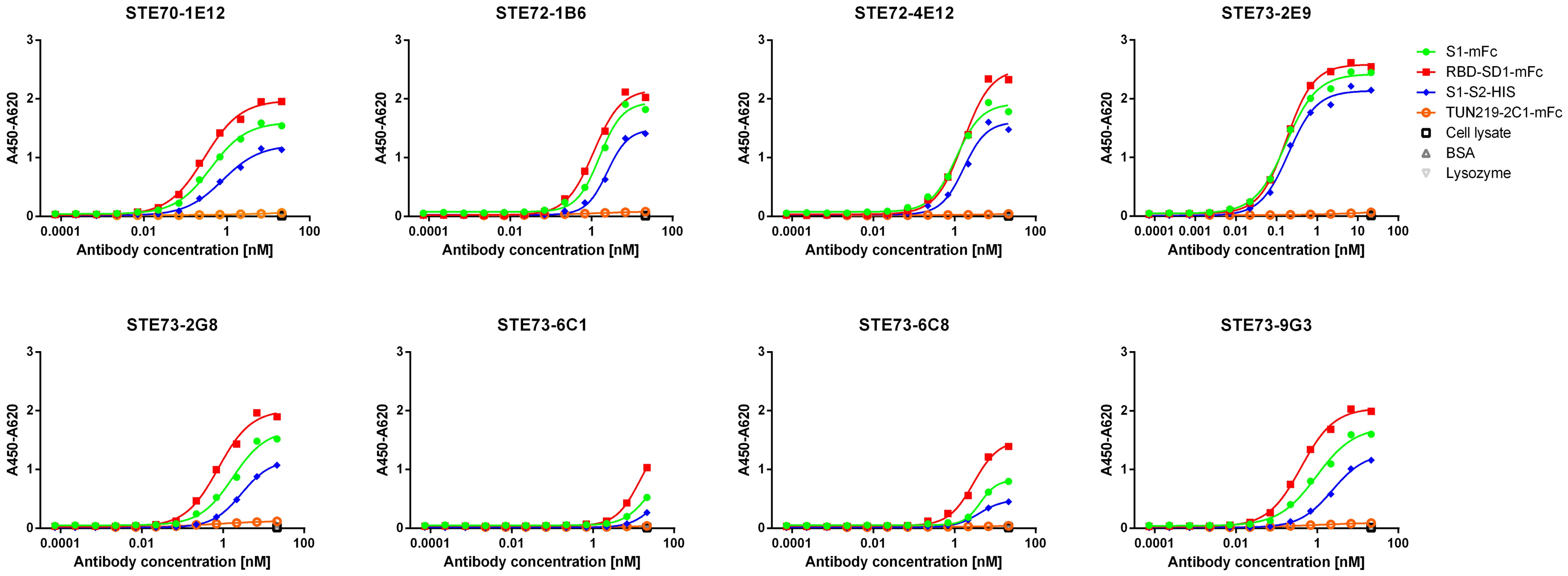

Supplementary Figure 8 Computational models of the antibodies (colored cartoon) on the spike trimer (surface, each monomer is in a different shade of grey). Only the variable region of the antibodies is shown.

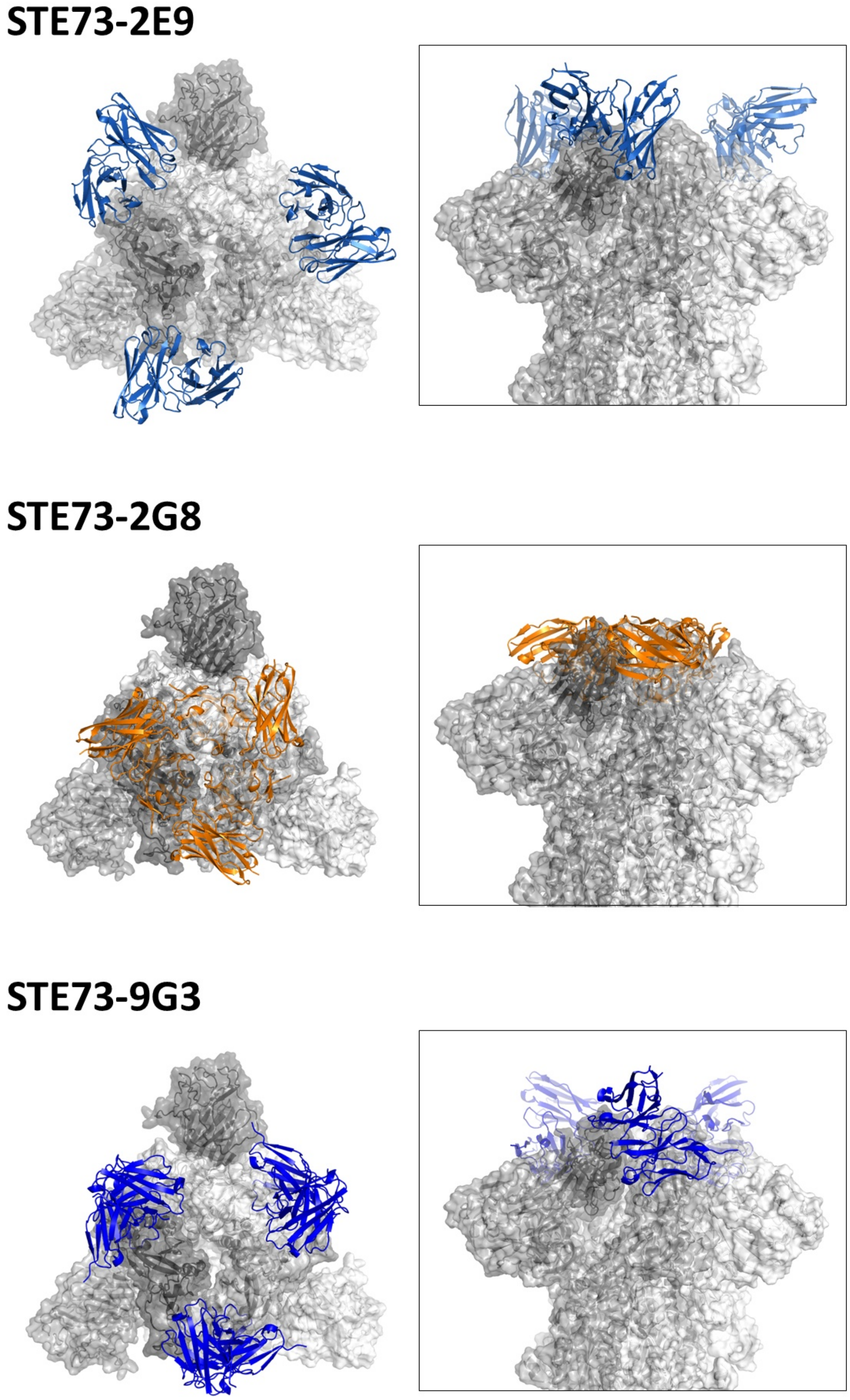

Supplementary Figure 9 SEC analysis of STE73-2E9 IgG under normal conditions (4°C, PBS, pH7.4), heat stress conditions (45°C for 24 h, PBS, pH7.4) and pH stress (4°C, 100 mM Na Acetate, pH3 for 24 h). Data shows a representative of two experiment.

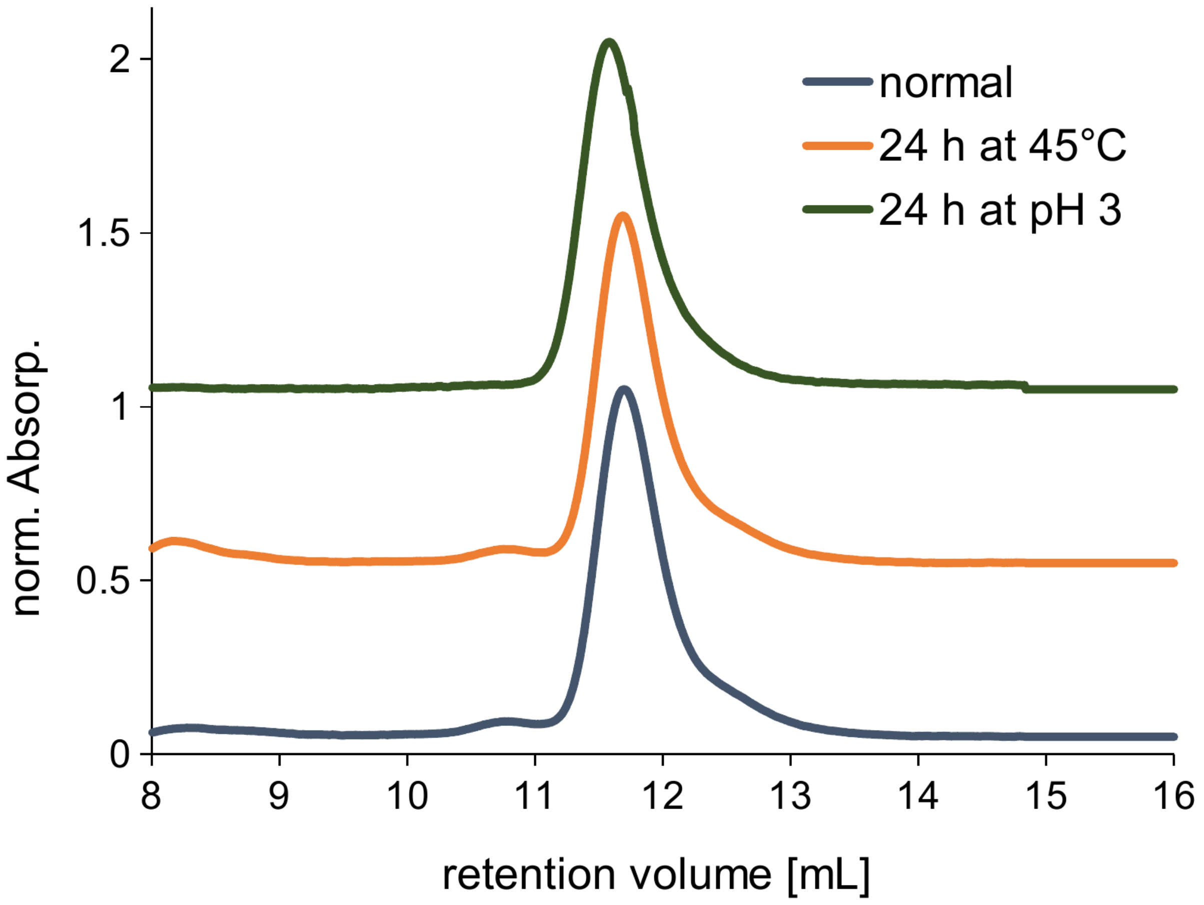

Supplementary Table 1 Overview oligononucleotide primers used in this study

primer name	sequence (5' > 3')	application
MBL-NcoI-RBD 32kDa_f	attaccatggcccgggtgcagcccac	cloning of RBD-SD1 and RBD-25
RBD-SD1-Not-rev	taatgcggccgcgctgcaaggggtgatg	cloning of RBD-SD1
GiR-RBD25kDa-NotI_r	taatgcggccgcgaagttcacgcatttgttcttc	cloning of RBD-25
Nco-S1-for	cattaccatggcccagtgcgtgaacc	cloning of S1 and S1-S2
S1-Not-rev (with furin)	ataatgcggccgcgcgcgcgcgtctggg	cloning of S1 with Furin
S1-Not-rev (without furin)	tggcggccgcggacgcagagccggggctgtttgtct	cloning of S1 without Furin
S1-S2-Not-rev	gcacgcgcggccgcctgttcatacttc	cloning of S1-S2
Nco-ACE-for	gcgtgccatggcccagtccaccattgaggaac	cloning of ACE2
ACE-extracell-Not-rev	cgcacgcggccgcggaaacagggggctgg	cloning of ACE2 extracellular domain
ACE-fulllength-Not-rev	cgcacgcggccgcaaaggaggtctgaac	cloning of ACE2 fulllength
V367F-for	ctactccttcctgtacaactccgccagcttc	insertion of mutation V367F
V367F-rev	gtacaggaaggagtagtcggccacgcaattgc	insertion of mutation V367F
V483A-for	cggcgcggaaggcttcaactgctac	insertion of mutation V483A
V483A-rev	cttccgcgccgttacaaggggtgc	insertion of mutation V483A
G476S-for	ggccagcagcaccccttgtaacggc	insertion of mutation G476S
G476S-rev	gctgctggcctgatagatctcggtggag	insertion of mutation G476S
N439K-for	cagcaagaacctggactccaaagtcggcgg	insertion of mutation N439K
N439K-rev	ccaggttcttgctgttccaggcaatcacacagc	insertion of mutation N439K
E484K-for	gcgtgaaaggcttcaactgctacttccc	insertion of mutation E484K
E484K-rev	gcctttcacgccgttacaaggggtg	insertion of mutation E484K
F486V-for	gaaggcgtcaactgctacttcccactgc	insertion of mutation F486V
F486V-rev	agttgacgccttccacgccgttacaagg	insertion of mutation F486V
G485R-for	gtggaacgcttcaactgctacttcccactgc	insertion of mutation G485R
G485R-rev	gaagcgttccacgccgttacaagggg	insertion of mutation G485R
OpiE2_for	cgcttatcgcgcctataaatac	sequencing of OpiE2 vector forward
IE1-rev	caaaaccccacaccaacaac	sequencing of OpiE2 vector reverse
ACE-seq-for	ccatgctaacggacccagg	internal sequencing of ACE2
S1Spike-seq-int-FBE-f	ccgaatccatcgtgcggttcc	internal sequencing of S1
S1+S2-int-S2-seq_F	gtggccagccagagcatcattgc	internal sequencing of S1-S2

ABC2	gtaacgtctctcagccaggtacagctgcagc	VH_f for cloning into pCSEHh1c.2 (STE73-6C8)
ABC4	gtaacgtctctcagccaggtgcaactgcagg	VH_f for cloning into pCSEHh1c.2 (STE73-9G3)
ABC5	gtaacgtctctcagccaggtgcagctggtg	VH_f for cloning into pCSEHh1c.2 (STE72-4E12)
ABC8	gtaacgtctctcagccaggtccagctggtg	VH_f for cloning into pCSEHh1c.2 (STE73-2E9, STE73-6B10, STE73-6C1)
ABC23	gtaacgtctctcggttcttctgagctgactcagg	VLλ_f for cloning into pCSL3hl.2 (STE73-2G8)
ABC28	gtaacgtctctcggtcagcttgtgctgactcaatc	VLλ_f for cloning into pCSL3hl.2 (STE73-6C1)
ABC34	tcctcgtctcagacctaggacggtgaccttgg	VLλ_r for cloning into pCSL3hl.2 (STE73-6C1, STE73-9G3)
ABC35	tcctcgtctcagacctaggacggtcagcttgg	VLλ_r for cloning into pCSL3hl.2 (STE73-2E9, STE73-2G8, STE73-6B10)
ABC40	gtaacgtctctcagccaggttcagctggtgc	VH_f for cloning into pCSEHh1c.2 (STE72-8A6, STE72-8A2)
ABC41	gtaacgtctctcggtcagcctgtgctgactcag	VLλ_f for cloning into pCSL3hl.2 (STE73-9G3)
ABC57	gtaacgtctctcggtcagtctgtgctgactcagc	VLλ_f for cloning into pCSL3hl.2 (STE73-2E9)
ABC59	gtaacgtctctcggtcagtctgccctgactcag	VLλ_f for cloning into pCSL3hl.2 (STE73-6B10)
ABC61	tcctcgtctcatagctgaggatacggtgacc	VH_r for cloning into pCSEHh1c.2 (STE70-1E12, STE72-8A6, STE72-8A2, STE72-4E12, STE72-1B6, STE73-2E9, STE73-2G8, STE73-6B10, STE73-6C1, STE73-6C8, STE73-9G3)
ABC63	gtaacgtctctcggtgaaacgacactcacgcagtc	VLk_f for cloning into pCSL3hk.2 (STE72-4E12)
ABC64	gtaacgtctctcggtgatattgtgatgacacagtctcc	VLk_f for cloning into pCSL3hk.2 (STE72-1B6)
ABC67	tcctcgtctcatacgtttgatttccaccttgg	VLk_r for cloning into pCSL3hk.2 (STE72-4E12)
ABC76	gtaacgtctctcggtgacatcgtgatgacccagtc	VLk_f for cloning into pCSL3hk.2 (STE72-8A6)
ABC107	tcctcgtctcatacgtttgatctccaccttgg	VLk_r for cloning into pCSL3hk.2 (STE72-8A2, STE72-1B6)
ABC110	tcctcgtctcatacgtttgatctccagcttgg	VLk_r for cloning into pCSL3hk.2 (STE72-8A6)
ABC113	tcctcgtctcagacctaggacggtcagtttgg	VLλ_r for cloning into pCSL3hl.2 (STE70-1E12, STE73-6C8)
GG_STE70-1E12_VH_f	gtaacgtctctcagccagatgcagctggtac	VH_f for cloning into pCSEHh1c.2 (STE70-1E12)
GG_STE70-1E12_VLl_f	gtaacgtctctcggtaattttatgctgactcagc	VLλ_f for cloning into pCSL3hl.2 (STE70-1E12)
GG_STE72-8A2_VLk_f	gtaacgtctctcggtgacatccggttgacccag	VLk_f for cloning into pCSL3hk.2 (STE72-8A2)
GG_STE73-2G8_VH_f	gtaacgtctctcagccaggtccagctggtacaatc	VH_f for cloning into pCSEHh1c.2 (STE73-2G8)
GG_STE73-6C8_VLl_f	gtaacgtctctcggtaattttatgctgactcaac	VLλ_f for cloning into pCSL3hl.2 (STE73-6C8)
GG_STE72-1B6_VH_f	gtaacgtctctcagccaggtgaagctggtacagtc	VH_f for cloning into pCSEHh1c.2 (STE72-1B6)
